# Spatial Discrimination Limit Analysis of Macrophage Phagocytosis Between Target Antigens and Non-Target Objects Using Microcapillary Manipulation Assay

**DOI:** 10.3390/mi15111394

**Published:** 2024-11-18

**Authors:** Maiha Ando, Dan Horonushi, Haruka Yuki, Shinya Kato, Amane Yoshida, Kenji Yasuda

**Affiliations:** 1 Department of Pure and Applied Physics, Graduate School of Advanced Science and Engineering, Waseda University, 3-4-1 Okubo, Shinjuku, Tokyo 169-8555, Japan; maiha.0105@akane.waseda.jp (M.A.); dan-horonushi@fuji.waseda.jp (D.H.); y.haru.13821@akane.waseda.jp (H.Y.); neersener0420@asagi.waseda.jp (A.Y.); 2 Department of Physics, School of Advanced Science and Engineering, Waseda University, 3-4-1 Okubo, Shinjuku, Tokyo 169-8555, Japan; sean0816@fuji.waseda.jp

**Keywords:** phagocytosis, macrophage, antibody-dependent cell phagocytosis, zipper mechanism, IgG-opsonized polystyrene particles, microcapillary manipulation assay, spatial discrimination limit

## Abstract

During phagocytosis, the FcGR–IgG bond is thought to be necessary to promote cell-membrane extension as the zipper mechanism. However, does this zipper mechanism provide a spatial antigen discrimination capability that allows macrophages to selectively phagocytose only antigens, especially for clusters with a mixture of antigens and non-antigens? To elucidate the ability and limitation of the zipper mechanism, we fed a coupled 2 μm IgG-coated and 4.5 μm non-coated polystyrene bead mixtures to macrophages and observed their phagocytosis. Macrophage engulfed the mixed clusters, including the 4.5 μm non-coated polystyrene part, indicating that the non-coated particles can be engulfed even without the zipper mechanism as far as coupled to the opsonized particles. In contrast, when the non-opsonized particle part was held by the microcapillary manipulation assay, macrophages pinched off the non-coated polystyrene particle part and internalized the opsonized particle part only. The results suggest that (1) an IgG-coated surface is needed to anchor phagocytosis by cell-membrane protrusion; however, (2) once the antibody-dependent cell phagocytosis is started, phagocytosis can proceed with the uncoated objects as the followers of the internalizing opsonized particles even without the support of the zipper mechanism. They may also indicate the concern of misleading the immune system to target unexpected objects because of their aggregation with target pathogens and the possibility of new medical applications to capture the non-opsonized target objects by the aggregation with small antigens to activate an immune response.

## 1. Introduction

Macrophages are a type of leukocyte and the first responder for invading antigens in the innate immune system in multicellular organisms [1,2,3,4,5,6,7,8], playing an important role in the initial stage of immune responses to recognize foreign substances such as bacteria (microorganisms) [9,10,11,12,13,14] and particulate matter 2.5 (PM_2.5_) [15,16,17] as antigens and phagocytose them. Based on internalized antigen information, macrophages activate other immune cells, including those of the acquired immune system, by antigen presentation [18,19] and cytokine secretion [20,21,22]. Therefore, elucidating the mechanism of antigen recognition by macrophages, i.e., how they discriminate between their targets and non-targets for phagocytosis, will lead to identifying the pathogenic process of autoinflammatory diseases and the immune escape mechanism of cancer cells.

Among the various reported mechanisms of antigen recognition followed by phagocytosis, the zipper mechanism proposed by Griffin et al. has been widely studied [23,24,25]. According to the zipper mechanism, macrophages initiate their phagocytosis by recognizing ligands on the antigen surface via receptors on the cell surface, and continuous receptor–ligand bond formation is required to progress the antibody-dependent cell phagocytosis [23,24]. Extensive studies in previous reports have supported the zipper mechanism and identified the types of receptors on macrophages and their ligands; for example, Fc receptors (FcR), recognizing the Fc region of immunoglobulins (antibodies), and complement receptor 3 (CR3), recognizing C3b, a degradation product of the third component of complement [18,26,27,28,29,30,31]. This set of receptors and ligands also affects the process of antigen internalization during phagocytosis. For example, Fc receptor-mediated phagocytosis involves four steps: antigen recognition by initial binding formation with the ligand, antigen engulfment by cell-membrane protrusion, phagocytic cup closure at the end of the engulfment, and retraction of the engulfed antigen [32,33,34]. In contrast, in complement receptor-mediated phagocytosis, the antigen is phagocytosed as it sinks into the cell after antigen recognition [25,35]. The intracellular proteins engaging in each step and the signaling cascades among them have also been elucidated [9,36,37].

As described above, the molecular biological and physiological mechanisms of antigen recognition and phagocytosis based on the zipper mechanism have been well studied. However, the limitations of the spatial discrimination ability of the zipper mechanism and its mechanistic mechanism are still unclear. Eisentraut et al. demonstrated that the spatial resolution of antigen recognition by macrophages is 450 nm in the experiment in which two opsonized microparticles were simultaneously brought into contact with one macrophage cell at various distances [38]. Griffin’s group prepared the half-opsonized erythrocytes , exploiting the depression in antigen internalization function of macrophages at low temperature and demonstrated that macrophages did not extend their membrane to the non-opsonized region of the prepared erythrocytes even when the temperature was restored [23,24]. The internalization phenomenon, which does not require the zipper mechanism, was also reported. For example, the cell membranes overcome and extend on the non-opsonized region of antigens by a mechanical factor of spontaneous closure of membranes as the general endocytosis process such as membrane remodeling by GTPase dynamine [39]. Gao et al. reported the Jurkat T-cell engulfment of Janus particles having both a BSA-passivated hemisphere and an anti-CD3-opsonized one [40]. They concluded that a combination of zipper and trigger mechanisms might endocytose the Janus particles in their study. However, what factors during the endocytosis actuate the trigger mechanism remains unspecified, and how the cell-membrane extension on the non-opsonized region is driven mechanically.

Here, in this study, we investigated the spatial recognition limits of macrophage phagocytosis when non-opsonized objects are bound to opsonized targets as coupled clusters. For that, we used the clusters of IgG-coated 2 μm polystyrene microparticles and non-coated 4.5 μm polystyrene microparticles mixtures aggregated by hydrophobic bonds and observed macrophage phagocytosis to verify whether the zipper mechanism can discriminate non-opsonized particles from opsonized particles in the mixed clusters. Furthermore, we elucidated the mechanical mechanism of internalization of ligand-deficient substances based on the displacement of clusters during phagocytosis and the retention or dissociation of the bonds between opsonized and non-opsonized particles. Finally, we confirmed the robustness of the zipper mechanism against ligand blanks with the observation of the macrophage membrane extension on clusters consisting of a single opsonized 4.5 μm polystyrene microparticle with multiple non-opsonized 2 μm polystyrene microparticles. In these experiments, we exploited a microcapillary manipulation assay that combines a microcapillary tube whose inner diameter of 2.5–4 μm and a pneumatic injector to enable strict control of the attachment orientation of the coupled two particles and their holding.

## 2. Materials and Methods

### 2.1. Cells

We used a mouse-derived macrophage-like cell line (J774.2; Sigma–Aldrich, St. Louis, MO, USA) as the macrophage model. The cells were grown at 37 °C under 5% CO_2_ in Dulbecco’s modified Eagle’s medium (DMEM: Gibco Thermo Fisher Scientific, Waltham, MA, USA) supplemented with 10% heat-inactivated fetal bovine serum (FBS: Gibco Thermo Fisher Scientific, Waltham, MA, USA) and 100 U/mL penicillin-streptomycin (Gibco Thermo Fisher Scientific, Waltham, MA, USA). Considering the change in characteristics depending on the number of cell passages, the passages were made up to the fifth generation.

### 2.2. Preparation of IgG-Coated Antigen Samples

The IgG-opsonized microparticles were prepared using the methods described in our previous reports [41,42]. In brief, first, 2 × 10^6^ of 2 μm polystyrene microparticles (19814-15; Polysciences, Warrington, PA, USA) were washed with phosphate-buffered saline (PBS; Takara Bio, Shiga, Japan) three times with centrifugation. Then, the microparticles were incubated in 10 mg/mL bovine serum albumin (BSA; Sigma–Aldrich, St. Louis, MO, USA) in PBS overnight at room temperature. After rewashing with PBS three times, the microparticles were then incubated in 0.33 mg/mL anti-albumin IgG (A10-127A; Bethyl Laboratories, Montgomery, TX, USA) in PBS for one hour at room temperature. Finally, the microparticles were washed with PBS, and their IgG opsonization was verified using a fluorescent secondary goat-antirabbit antibody (Alexa Fluor 488; Abcam, Cambridge, UK). As the non-opsonized microparticles, we used 4.5 μm microparticles cultivated in and washed with PBS for the same time as the IgG-opsonized microparticles to align their experimental conditions except for BSA and IgG modification.

Then, we prepared IgG-opsonized and non-opsonized polystyrene clusters (cluster antigen) by hydrophobically aggregating 2 μm opsonized polystyrene microparticles and 4.5 μm non-opsonized polystyrene microparticles, based on Yi’s method [43]. In brief, opsonized microparticles and non-opsonized microparticles were dispersed in hexane (Fujifilm Wako Pure Chemical, Osaka, Japan), and PBS was added and mixed sufficiently to disperse microparticles. The microparticle mixture in PBS-hexane solvent was dripped on a cultivation dish (AGC Techno Glass, Shizuoka, Japan) and dried until evaporated completely. Just after drying, PBS was added to the dish, and the formed clusters of mixed microparticles were collected by pipetting. Finally, the collected clusters were washed with PBS three times with centrifugation and stored in PBS at 4 °C. The IgG-opsonized microparticles and non-opsonized microparticles in the clusters were identified by their diameter differences.

### 2.3. Observation of Cluster Antigen Phagocytosis

We observed the cluster antigen phagocytosis by macrophages with microcapillary manipulation assay exploiting the microparticle manipulation of glass-microcapillary tubes whose inner diameter is 2.5–4 μm. The microcapillary tubes were fabricated as follows: one mm-glass tube (GD-1; Narishige, Tokyo, Japan) was torn off with a micropipette puller (P-97; Sutter Instrument, Novato, CA, USA), and their inner diameter was adjusted and their head portion was bent 30° to be parallel with the bottom of the culture dish when attached to a micromanipulator, with a microforge (MF-2; Narishige, Tokyo, Japan). Using this microcapillary tube, we fed cluster antigens to single macrophages.

Observation procedure was as follows; first, 5.0 × 10^4^ macrophages (J774.2) were placed in a 35 mm glass base dish (AGC Techno Glass, Shizuoka, Japan) with 2 mL DMEM and stabilized in an incubator (MCO-5M; SANYO Electric, Osaka, Japan) at 37 °C under 5% CO_2_ for 3 h. Next, the DMEM was replaced with CO_2_ independent medium (Gibco, Thermo Fisher Scientific, Waltham, MA, USA), and 1.0 × 10^4^ cluster antigens were added mildly to the stabilized dish. Then, the dish was set on the stage of the inverted microscope (IX70, 100× oil immersion objective lens; Olympus, Tokyo, Japan) in which the temperature was controlled at 37 °C with a heater (IX-CBIB100, Olympus, Tokyo, Japan) and an acrylic insulation box. One cluster antigen was captured and brought into contact with a single cell adhered on the bottom of the culture dish with the suction of microcapillary tubes connected with a pneumatic injector (Celltram 4r Air; Eppendorf, Hamburg, Germany) and a 3D electric micromanipulator (MP-285; Sutter Instrument, Novato, CA, USA). The phagocytic process was recorded in time-lapse images at 5 s intervals with a digital camera (1500M-GE; Thorlabs, Newton, NJ, USA).

## 3. Results and Discussion

### 3.1. Engulfment of Cluster of IgG-Coated/Non-Coated Polystyrene Particle Mixture

**Figure 1:**
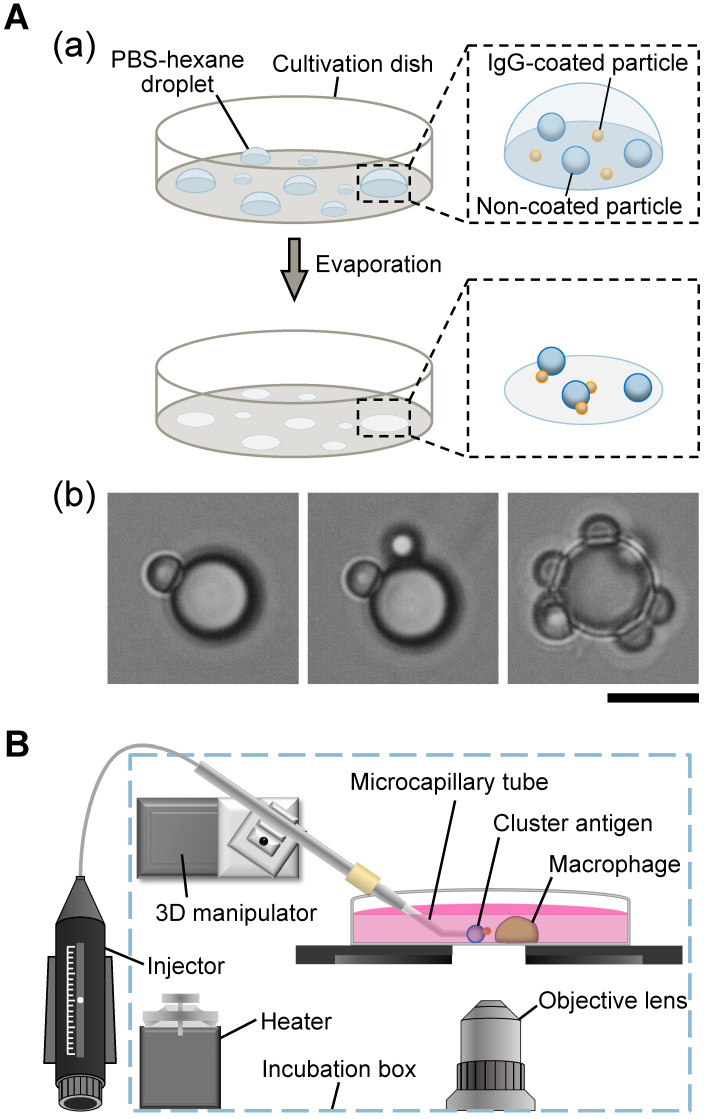
Preparation of clusters of opsonized/non-opsonized polystyrene particle mixture and microcapillary manipulation assay for observation. (**A**): Preparation of clusters of IgG-coated (small yellow) and non-coated (large blue) polystyrene microparticle mixtures. (**a**) Schematics of cluster formation procedure exploiting hydrophobic aggregation. (**b**) Micrographs of 2 μm opsonized and 4.5 μm non-opsonized polystyrene microparticle clusters. Bar, 5 μm. (**B**): Schematics of experimental equipment for microparticle cluster phagocytosis observations (microcapillary manipulation assay). The microcapillary tube mounted in the 3D manipulator and connected with the pneumatic injector captured each cluster and brought it to the macrophage for phagocytosis observation.

First, we prepared the clusters of opsonized and non-opsonized polystyrene microparticles as described in Figure 1A(a) Two different sizes of polystyrene particles were prepared for clustering: 2 μm opsonized and 4.5 μm non-opsonized polystyrene microparticles. The difference in these two particle sizes was used to identify the type of each particle. A mixture of two types of particles was diluted in a PBS-hexane medium and placed on the cultivation dish as droplets. The solution was evaporated in a vacuum, and the particle clusters agglomerated in this process were used in the experiment. As shown in Figure 1A(b), clusters of coupled microparticle mixtures were formed, and we picked up and transported each cluster to the macrophages with a microcapillary manipulation assay (Figure 1B).

**Figure 2:**
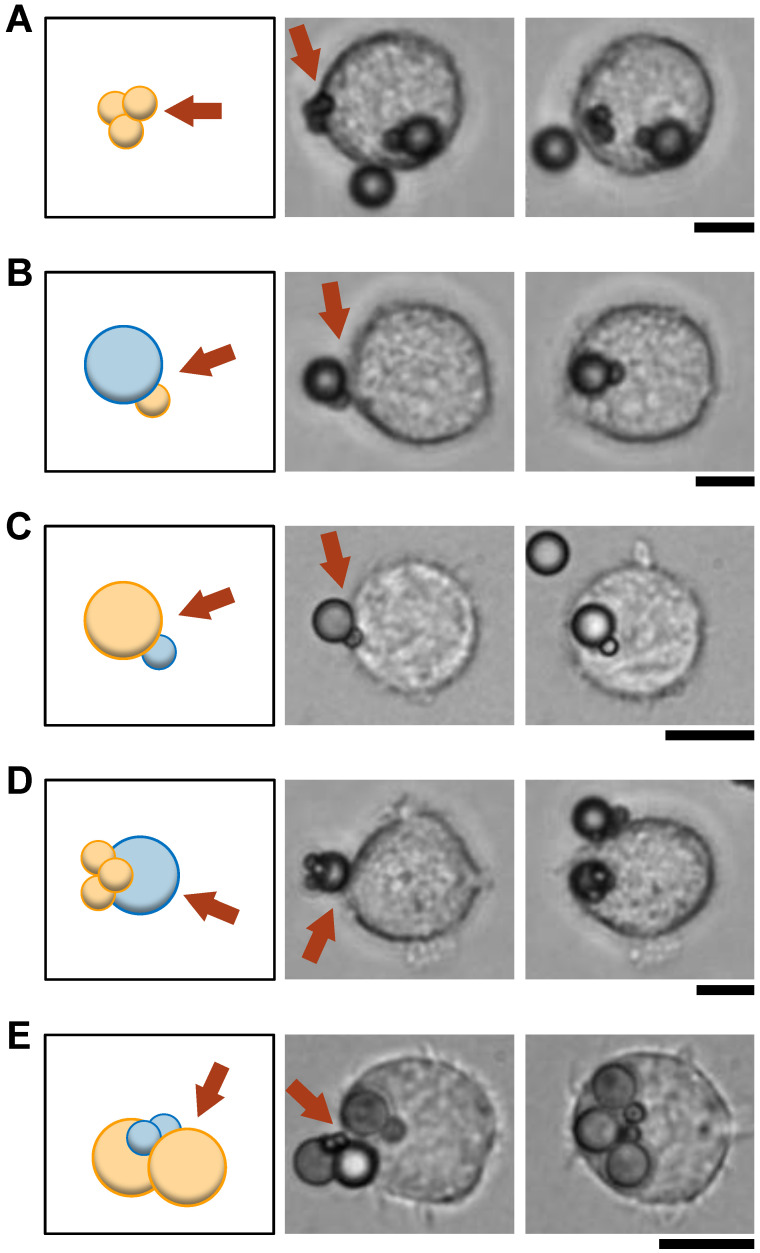
Evaluation of IgG-coated/non-coated polystyrene microparticle mixture. **Left**: Schematics of clusters. The yellow and blue particles represent the IgG-coated and non-coated particles, respectively. The red arrows in the schematics point to the initial contact point with macrophages (also shown in the center micrographs). **Center** and **Right**: Micrographs of just after clusters attached and internalized by macrophages, respectively. Bars, 10 μm. (**A**): A cluster consisting of three 2 μm IgG-coated particles. (**B**): A coupled two-particle cluster consisting of a 2 μm IgG-coated particle and a 4.5 μm non-coated particle. (**C**): A coupled two-particle cluster consisting of a 4.5 μm IgG-coated particle and a 2 μm non-coated particle (reversed particle sizes in Figure (**B**)). (**D**): A four-particle cluster consisting of three 2 μm IgG-coated particles and a 4.5 μm non-coated particle. (**E**): A four-particle sandwich-like cluster consisting of two 4.5μm IgG-coated particles at both ends and two 2 μm non-coated particles in between.

Figure 2 shows the results of engulfment of clusters of IgG-coated/non-coated particle mixtures. As shown in Figure 2A, when a 2 μm IgG-coated three polystyrene bead cluster (an orange small three particle cluster in the schematic) was attached on the left side of the macrophage, the cluster was internalized maintaining their clustered form, whereas the single non-coated bead was not engulfed even though it was attached on the lower side of the macrophage at the same time. Figure 2B shows the result of the coupled 2 μm opsonized (orange in the schematic) and 4.5 μm non-opsonized (blue in the schematic) two-particle mixture for engulfment. As shown in the micrographs, both particles in the cluster were engulfed regardless of the opsonization. It was also observed that opsonized and non-opsonized two-particle mixtures with exchanged particle sizes were phagocytosed, indicating that the exchange of particle size did not affect the results (Figure 2C). Furthermore, when the cluster adhered to the macrophage from the 4.5 μm non-opsonized particle side, both the opsonized three particles and the non-opsonized particle in the cluster were internalized into the macrophage while maintaining their bound shape (Figure 2D). Figure 2E shows the uptake of two non-opsonized small particles attached between two opsonized large particles. As shown in the micrographs, the four-particle cluster was internalized into the macrophage while retaining its bound form.

The zipper mechanism suggests that the non-opsonized microparticles are not engulfed because of the lack of ligand-receptor-bond formation on the surface of the non-coated microparticles. However, the results indicate that non-opsonized particles attached as mixed clusters to opsonized particles were phagocytosed simultaneously. There are at least two possibilities to explain this. One is that the free IgG was attached to the non-coated microparticles during the cluster formation process. Another is that the zipper mechanism has a redundancy, enabling macrophages to engulf non-coated microparticles coupled to the IgG-coated microparticles.

To clarify and explain the internalization mechanism in this engulfment described above, we need to measure it in a controlled arrangement of the cluster orientation.

### 3.2. Controlled Attachment of Coupled IgG-Coated/Non-Coated Polystyrene Two Particles with Microcapillary Tube Manipulation: Vertical Direction

We designed a two-step, fully direction-controlled attachment of the coupled IgG-coated/non-coated polystyrene two particles to overcome the limitation of the above random cluster phagocytosis experiment. In the first step, the non-coated particle side of the coupled two particles was contacted to the surface of single macrophages to confirm that its surface had not been opsonized (step 1). We regarded its surface as “non-opsonized” when the contacted particle did not attach to the cell membrane and was not engulfed within 10 min after their contact because the results of our previous report showed that the sum of the mean phagocytosis time against 2 μm and 4.5 μm opsonized polystyrene particles was 5 min [41]. If the non-coated particle was engulfed, we excluded this sample to avoid the potential IgG contamination on the surface of the non-coated microparticles. However, we have not observed any non-coated particle side of the cluster engulfed in this step during the entire experiment, indicating no free IgG was attached to the non-coated particles during our experiment. After this confirmation, we released and inverted the direction of the coupled two-particle cluster to face the opsonized particle side that can attach to the macrophage. Then, we attached the opsonized particle side of the coupled two-particle clusters from the vertical direction and observed the phagocytic response of clusters (step 2).

**Figure 3:**
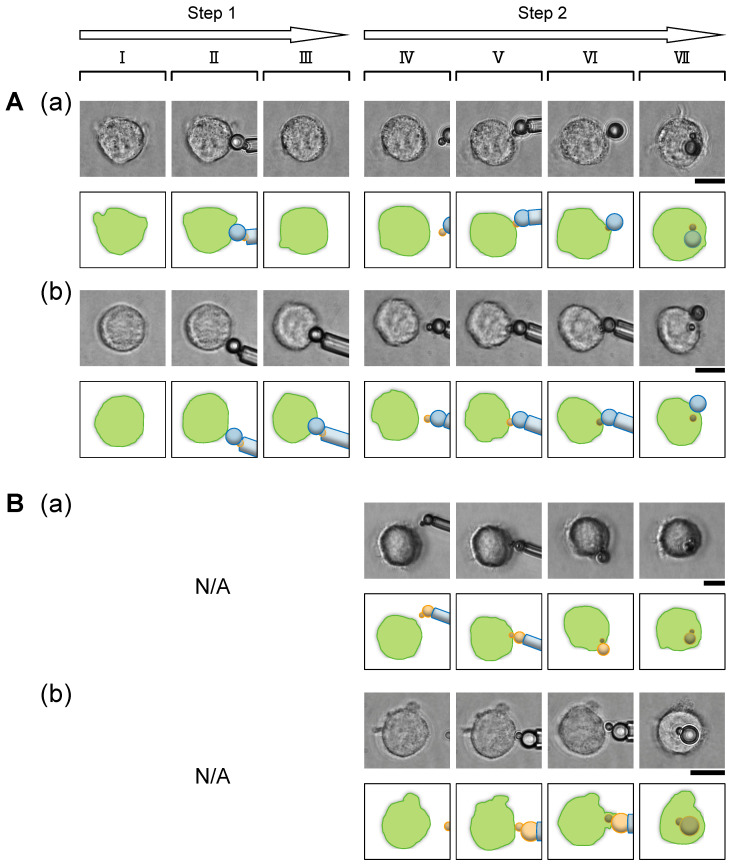
Vertical contact of the coupled two particles. **Step 1**: Confirmation of non-opsonization of non-coated particles. **I** and **II**, macrophages before and just after contact with the non-coated particle side of the cluster, respectively. **III**, macrophages 10 min after the contact started. In (**A**(**a**)), the micrograph was taken just after the particles detached from the macrophage surfaces without any resistance by adhesion. **Step 2**: Contact of the cluster from the IgG-coated particle side. **IV** and **V**, macrophages before and just after contact with the IgG-coated particle side of the cluster, respectively. **VI**, completion of engulfment for 2 μm IgG-coated particle side of the cluster. **VII**, completion of phagocytic responses. Upper, micrographs; Lower, schematics of micrographs. Bars, 10 μm. (**A**): Vertical contact of the coupled IgG-coated/non-coated polystyrene two particles. (**a**) A two-particle cluster was vertically attached to the macrophage with microcapillary manipulation. After confirming that the cluster adhered to the macrophages, the cluster was released. (**b**) The non-coated particle side of the cluster was held by the microcapillary manipulator throughout step 2, in contrast to (**A**(**a**)). (**B**): Vertical contact of the coupled two IgG-coated polystyrene particles. Here, step 1 was waived because both particles were coated with IgG. (**a**) A two-particle cluster was vertically attached to the macrophage with the microcapillary manipulator. After confirming that the cluster adhered to the macrophages, the cluster was released. (**b**) The larger particle was held by the microcapillary manipulator throughout step 2, in contrast to (**B**(**a**)).

Figure 3 shows the results. After the coupled two-particle clusters were attached to the macrophage from the IgG-coated particle side with the microcapillary manipulator and were released, we found that the macrophage phagocytosed the entire coupled two particles, including the non-coated microparticles (Figure 3A(a)). Interestingly, during phagocytosis of the non-coated particle side, the macrophages internalized the particle as it sinks into the cell rather than the cell-membrane protrusion.

To verify whether this dragging is an essential process for macrophages to phagocytose the non-coated particles, we kept holding the non-coated particles of the coupled two-particle clusters with a microcapillary manipulator even after the cell-membrane extended to the entire part of the opsonized particle side of the cluster in step 2 and checked whether the holding of the non-coated part of the coupled two-particle clusters prevents phagocytosis of the clusters. As shown in Figure 3A(b), we observed that the macrophage engulfing the IgG-coated particle of the coupled two particles tore the bond between them and internalized only the IgG-coated particle. This result indicates that macrophages protrude their membrane and engulf antigens when ligand-receptor bonds can be formed on them (i.e., the zipper mechanism is valid), whereas they stop their spontaneous membrane extension and start to drag the engulfing antigen into them even though their phagocytic cup is not completely closed when their receptors become unable to bind with the next ligand during the engulfment. However, two possible causes remain: the looseness of bonds between the IgG-coated particle and the non-coated one in the coupled two particles and the geometrical shape discontinuity in the coupling part of the two particles.

To test the above two possibilities, we prepared the coupled two-particle cluster in which both were optimized, and the other conditions were the same. We fed these coupled particles to single macrophages and observed that they phagocytosed the entire particles without pinching off the coupled bond regardless of the hold of the microcapillary manipulation (Figure 3B(a,b)). From these results, we can exclude the above two possibilities and conclude that the separation of the IgG-coated particle and the non-coated one was caused by the retraction of the IgG-coated one by the macrophages.

These results suggest that (1) macrophages stop their further spontaneous phagocytic cup progression and shift to the antigen retraction stage, when they become unable to form ligand-receptor bonds during phagocytosis as the zipper mechanism, (2) the retraction occurs to internalize the formed ligand-receptor bonds and only the opsonized part of the two-particle cluster is internalized to finish the uptake process as long as the macrophages can tear off the non-opsonized part from the engulfed particle part, and (3) otherwise, the entire substance including non-opsonized regions sunk into the cell following to the internalization of the opsonized part. The suggestion described in (2) is particularly interesting in that macrophages phagocytose the non-opsonized particle not because they overlooked its ligand deficiency but because they could not remove it at the border with the IgG-coated particle. Although macrophages obey the zipper mechanism, they may also have the flexibility to switch to other mechanisms (e.g., mechanical mechanisms such as antigen retraction) to maintain their phagocytosis against the antigens, which the macrophages cannot deal with only by the zipper mechanism. If the above hypothesis is correct, the spatial discrimination limit of macrophages for phagocytosis of the opsonized particles from the non-opsonized particles in their mixed clusters should be challenging to answer. For the mixture clusters, the ability of the phagocytic cup to pinch off the non-opsonized parts from the opsonized parts in the clusters should become an answer for that.

### 3.3. Controlled Attachment of Coupled IgG-Coated/Non-Coated Polystyrene Two Particles with Microcapillary Tube Manipulation: Horizontal Direction

Next, we attached the coupled two particles in the horizontal direction to the macrophage. As described above, macrophages may internalize all coupled particles by retracting the non-coated particle based on receptor–ligand binding on the IgG-coated particle. Then, how do macrophages respond to the attached cluster when the IgG-coted and non-coated particles of the cluster are brought into contact with the cell simultaneously? Considering our above suggestion, we expected that the macrophages phagocytosed the entire coupled particles from the IgG-coated particle side to the non-coated side stepwisely and that the retraction force caused some motions of the entire coupled particle clusters when the coupled particle clusters were brought into contact with the cell horizontally and then the microcapillary maniopulator released them. On the other hand, the coupled particles would be internalized as they sunk into the cell while maintaining their initial horizontal contact orientation if macrophages recognized the entire coupled particle clusters as a single target similar to the two close microbeads in Eisentraut’s experiments [38].

**Figure 4:**
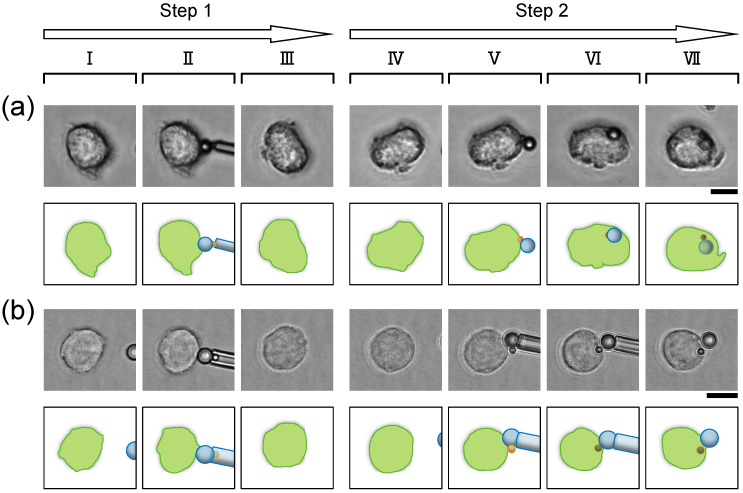
Horizontal contact of the coupled two particles. **Step 1**: Confirmation of non-opsonization of non-coated particle side of the cluster. **I** and **II**, macrophages before and just after the contact with the non-coated particle side of the cluster, respectively. **III**, macrophages 10 min after the contact started. In both (**a**) and (**b**), the micrographs were taken just after the particles detached from the macrophage surfaces without any resistance by adhesion. **Step 2**: Contact of IgG-coated particle side of the cluster. **IV** and **V**, macrophages before and just after the contact with IgG-coated particles, respectively. **VI**, completion of engulfment for 2 μm IgG-coated particles. **VII**, completion of phagocytic responses. Upper, micrographs; Lower, schematics of micrographs. Bars, 10μm. (**a**) A two-particle cluster was horizontally attached to the macrophage with the microcapillary manipulator. After confirming that the cluster adhered to the macrophages, the cluster was released. (**b**) The non-coated particle side of the cluster was held by the microcapillary manipulator throughout step 2, in contrast to (**a**).

Figure 4a shows the engulfment of the coupled two-microparticle cluster after it was attached to the macrophage with the microcapillary manipulator. In this experiment, after confirming that the cluster adhered to the macrophages, the cluster was released. The macrophage phagocytosed the coupled two-microparticle cluster stepwise, from the IgG-coated particle to the non-coated one. This result implies the predominance of the zipper mechanism; macrophages preferentially phagocytose opsonized antigens even when the opsonized antigens and non-opsonized objects are in close contact with the macrophages.

We also carried out the same horizontal contact while holding the non-coated particles by the microcapillary manipulator to confirm whether the internalization of the coupled particles had been induced by the retraction of IgG-coated particles. In the phagocytosis process shown in Figure 4a, we did not observe obvious motions of the entire coupled particles, which the retraction of the IgG-coated particle might cause. Then, we held the non-coated particles by the microcapillary manipulator and confirmed whether the retraction force tore off the IgG-coated particle from the non-coated one. As shown in Figure 4b, the macrophage separated the coupled two particles and phagocytosed only the IgG-coated particles, although the non-coated particle was kept in contact with the cell. From this result, we can say that the retraction of IgG-coated particles works in phagocytoses against the coupled particles regardless of their initial contact orientation.

### 3.4. Pinch-Off Force of Phagocytosis at the End of the Uptake Process

The above results suggest that the internalization of the entire coupled particles or separating the particle couplings followed by the phagocytosis of only IgG-coated particles is caused by mechanical retraction of IgG-coated particles by macrophages. Then, the next question is the phagocytic responses when the macrophage encounters small non-coated particles during the engulfment of a large IgG-coated particle. In the experiments described above, since the IgG-coated particles were smaller than the non-coated particles, the macrophages had completed the formation of receptor–ligand bonds on the IgG-coated particle and could not continue the phagocytosis based on the zipper mechanism in principle, when they encountered the non-coated particle. On the other hand, when the IgG-coated particles are large, the receptors on macrophages can bind with the ligands around the non-coated particles when the macrophages encounter the non-coated particles so that the non-coated particles are dealt with while the phagocytosis based on the zipper mechanism continues. There are two possible phagocytic behaviors based on the above results and their implications. First, according to the zipper mechanism, IgG-coated particles would continue to be engulfed, eliminating the non-coated particles. The other is that macrophages perceive the blank of ligands on the antigens sensitively and begin antigen retraction, resulting in the internalization of the entire coupled particles, including the non-coated particles. To verify this question, we prepared the cluster antigens consisting of an IgG-coated 4.5 μm particle bound with surrounding non-coated 2 μm particles and fed the cluster antigens to single macrophages.

**Figure 5:**
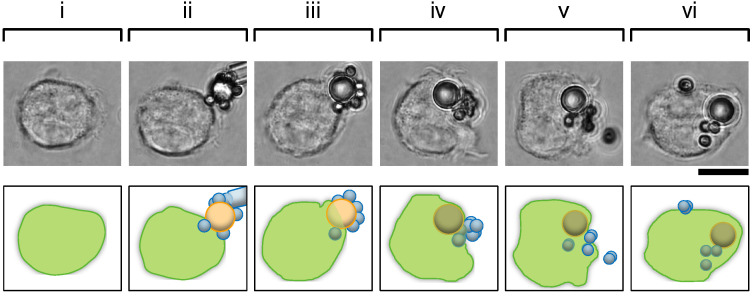
Gathering of non-opsonized particles surrounding an opsonized particle during phagocytosis. Upper, micrographs; Lower, schematics of micrographs. Bar, 10 μm. (**i**,**ii**), a macrophage before and just after the contact of the cluster, respectively. (**iii**), engulfment of the opsonized particle part of the cluster. The non-coated 2 μm particles surrounding the opsonized particle were swept backward by the cell membrane. (**iv**), completion of internalization of the opsonized particle part followed by the internalization of gathered non-opsonized particles behind the opsonized particle. (**v**), pinching off non-internalized non-coated particles. Two non-internalized, non-coated particles gathered at the end of the phagocytic cup were pinched off and left the cell or the cluster completely. (**vi**), completion of phagocytic responses.

Figure 5 shows the phagocytosis result of the mixture of opsonized and non-opsonized polystyrene particles. As shown in the micrographs, non-coated 2 μm polystyrene particles attached surrounding an IgG-coated 4.5 μm polystyrene particle. When we attached this cluster to a macrophage, internalization started, and the non-opsonized particles were wiped backward from the surface of the opsonized particle to the behind of the opsonized particle. At the end of the uptake process, the IgG-coated 2 μm particle and three non-coated particles were internalized. However, the other non-coated particles were not internalized and remained gathered. Finally, the cellular response was finished after all the non-internalized, non-coated particles had been pinched off.

This supports the results of the vertical coupled IgG-coated/non-coated polystyrene two-particle internalization. When the cell membrane proceeded on the opsonized particle as the zipper mechanism, non-opsonized particles were torn off from the opsonized particle, causing the gathering of non-opsonized particles backward from the opsonized particle. Then, these non-opsonized particles were pinched off; otherwise, they were dragged into the cell with the opsonized particle, similar to the above coupled two-particle internalization.

These similarities imply the mechanism of non-opsonized particle internalization more clearly. The zipper mechanism recognizes the opsonized particle and proceeds only on the opsonized target surface while removing non-opsonized objects if necessary. However, at the final step of the uptake process, since the pinch-off force of the cell membrane, which is caused by the GTPase dynamine, is not sufficient to separate the non-opsonized particles from the opsonized targets completely, non-opsonized particles are internalized during the opsonized particle internalization as the coupled followers of the opsonized and non-opsonized-particle train.

### 3.5. Spatial Discrimination Limit of Macrophage Phagocytosis

As described above, the controlled two-step phagocytosis experiment of the coupled two-particle clusters confirmed that the non-coated particles are internalized following the engulfment of the IgG-coated particles when coupled to the IgG-coated particles, even though they are attached as aggregation. The coupled two particles were engulfed from their opsonized particle regardless of their initial contact orientation, indicating the predominance of the zipper mechanism. Our microcapillary manipulation assay demonstrated the necessity of mechanical retraction of the engulfed opsonized antigen in the following internalization of the coupled non-opsonized objects.

Then how does this serial internalization proceed in macrophages? There are at least two types of internalization of opsonized particles. One is the Fc receptor (FcR)-based zipper mechanism, and the other is complement receptor (CR)-mediated phagocytosis [25,32,33,34,35]. If the internalization pathway of the CR-mediated phagocytosis can be triggered after the initiation of FcR-based phagocytosis or can be coupled to the FcR-based phagocytosis when the FcR zipper mechanism process cannot pinch off the phagosome from the cell membrane by the GTPase dynamin (these marks the end of the uptake process), the non-opsonized objects can be internalized by the internalization pathway of the CR-mediated phagocytosis as long as they are coupled with a opsonized antigen and the zipper mechanism works first. Michl et al. reported that Fc receptors and complement receptors work independently; even when one type of receptor is engaging in frustrated phagocytosis (spreading on opsonized substrates), the other type can engage in antigen phagocytosis [30]. Switching between the FcR-based zipper mechanism and the CR-mediated phagocytosis we described above seems to conflict with this report, but this switching may be triggered to continue phagocytosis only when one phagocytic mechanism becomes unavailable during phagocytosis against a single antigen.

Kress’s group estimated that the Fc receptor spacing is 0.30 μm in their analysis of the spatial resolution limit of phagocytosis [38], suggesting that the internalization of non-coated 4.5 μm particles cannot be explained only by the zipper mechanism. In this study, it was suggested that the mechanical antigen retraction mechanism began to work when the zipper mechanism became invalid and enabled macrophages to internalize the non-opsonized particles. The cell-membrane stall at the Janus boundary, observed in Gao’s experiment [40], might be a transitional period from the zipper mechanism to the mechanical mechanism. Although Griffin’s group showed their zipper mechanism by the experiment in which the temperature was raised from a low temperature to the optimum temperature for phagocytosis and the phagocytic activity of macrophages was restored [23,24], the ligand-deficient antigen might not be internalized because this mechanical antigen retraction mechanism was lost at low temperature.

Moreover, in our horizontal contact experiments, the IgG-coated particle was phagocytosed before the non-coated one, although these two particles were brought into close and simultaneous contact with the cell. Kress’s group estimated the spatial resolution of macrophage antigen recognition at 450 nm using two opsonized microbeads and demonstrated that the two close antigens within this resolution limit are internalized together. From these findings, it may be possible that the spatial resolution with one opsonized and one non-opsonized microbead is smaller than 450 nm.

The difference in results in two complementary experiments, with/without holding of non-coated microparticle during phagocytosis, also indicates the strength of internalization, which can separate the coupled two particles, and the strength of the GTPase dynamin, which could not pinch off the coupled two microparticles.

The results imply the possibility that the engulfment of non-target objects can occur easily when they are coupled to the target antigens as clusters. In our experiments, the size of IgG-coated particles was more than two times smaller than the non-coated particles. However, once the engulfment started by the smaller opsonized particles, the attached larger non-coated particles were engulfed. This evidence gives us caution and a bright side to these results for potential application. The caution is that the aggregation of antigens with a non-target host component may induce an unexpected immune response through the antigen presentation and pro-inflammatory process in macrophages; for example, endogenous ligands may be more phagocytosed with invading pathogens during infection, leading to auto-inflammations. The bright side of the results for a potential application is that particles like PM_2.5_ might be internalized into macrophages when the antigens are coupled with those unlabeled particles. As shown in the results, once the aggregation is formed, we can remove them with macrophages.

In this study, we found the engulfment of the cluster of IgG-coated microparticles and non-coated ones and suggested that the mechanical antigen retraction mechanism works complementary to the zipper mechanism from the view of the bond separation in clusters. To avoid unexpected opsonization onto the non-coated particles, we adopted a 2-step phagocytosis experiment: step 1 for confirmation of non-opsonization on the non-coated particles and step 2 for observation of the phagocytosis against clusters. Based on step 1, we concluded that the internalization of non-coated particles in step 2 occurred due to the coupling with IgG-coated particles. Moreover, exploiting the microcapillary manipulation assay enabled us to control the orientation of clusters precisely. In addition, by confirming the engulfment of the clusters consisting only of IgG-coated particles, we excluded the possibility of the loose bond in the prepared clusters and showed that the geometrical shape discontinuity at the border of the coupled particles did not affect our result.

Using a microcapillary tube manipulation assay, direction-controlled attachment of the coupled two microparticles to macrophages revealed that the non-opsonized polystyrene particles could be internalized into macrophages after the opsonized particle part in the coupled opsonized particles is engulfed. The results indicate two possibilities: the concern of misleading the immune system to target unexpected objects because of their aggregation with target pathogens and the possibility of new medical applications to capture the non-opsonized target objects by the aggregation with small antigens to activate an immune response. The next step is to elucidate the molecular mechanism of this phenomenon, which adds to the zipper mechanism, to translate the obtained results into practical applications in clinical immunology.

## 4. Conclusions

We imparted a linked IgG-coated and non-coated polystyrene bead combination to macrophages and watched their phagocytosis to clarify the zipper mechanism’s capabilities and limitations. Our study demonstrated that phagocytosis by cell-membrane protrusion, a process that requires an IgG-coated surface for anchoring, can also occur with uncoated objects as the followers of the internalizing opsonized particles, even in the absence of the zipper mechanism once antibody-dependent cell phagocytosis has begun. Notably, macrophages, one of the key players in antigen presentation, can inadvertently cause redundancy in the immune response system. On the other hand, the bright side of this discovery is that it may be possible to eliminate targets that evade attack from the immune system through immune tolerance by labeling them appropriately. 

## Data Availability

The original contributions presented in the study are included in the article, further inquiries can be directed to the corresponding author.
